# Generative Pre-trained Transformer-4 (GPT-4) generated psychological reports in psychodynamic perspective: a pilot study on quality of report, risk of hallucination, and client satisfaction

**DOI:** 10.1192/j.eurpsy.2025.1434

**Published:** 2025-08-26

**Authors:** T.-S. Kim, N. Kim, J. Lee

**Affiliations:** 1 Department of Psychiatry, Seoul St. Mary’s Hospital, The Catholic University of Korea, College of Medicine; 2 Department of Clinical Medical Sciences, Seoul National University, Seoul; 3 Department of Psychiatry, Uijeongbu Eulji Medical Center, Eulji University School of Medicine, Uijeongbu, Korea, Republic Of

## Abstract

**Introduction:**

Recently, there has been growing interest in leveraging large language models (LLMs) in psychiatry and counseling. Specifically, there is a need to develop LLM-based programs that generate psychodynamic assessments, helping individuals gain self-insight and evaluate the quality of such services. However, research in this area remains limited.

**Objectives:**

This pilot study aims to evaluate quality, risk of hallucination, and client satisfaction with psychodynamic psychological reports generated by GPT-4.

**Methods:**

The reports consisted of five components: psychodynamic formulation, psychopathology, parental influence, defense mechanisms, and client strengths. Participants experiencing distress from recurring interpersonal issues were recruited for the study, which followed three stages: 1) GPT-4 generated tailored questions for participants to infer psychodynamic formulations, then used the responses to create psychological reports. 2) Seven psychiatry professors from various university hospitals assessed the reports for quality and hallucination risk, comparing GPT-4-generated reports with expert-inferred reports. 3) Participants evaluated their satisfaction with the psychological reports. All assessments were conducted using self-report questionnaires based on a Likert scale developed for this study.

**Results:**

Ten participants were recruited for the study, with an average age of 32 years. The median response indicated that the quality across all five components of the psychological report aligned closely with expert evaluations. The risk of hallucination was assessed to be minimal, ranging from unlikely to minor. In the satisfaction evaluation, over 90% of participants agreed or strongly agreed that the report was clear, insightful, credible, useful, satisfying, and recommendable.

**Conclusions:**

These findings suggest that artificial intelligence may have the potential to provide expert-level psychodynamic interpretations with minimum face-to-face interaction.

**Disclosure of Interest:**

None Declared

